# Keeping the heart empty and beating: an alternative technique to preserve hypertrophied hearts during valvular surgery

**DOI:** 10.1186/s13019-015-0273-2

**Published:** 2015-05-13

**Authors:** Shangdian Liu, Zonghong Liu, Lulu Li, Pengfei Liu, Hongyu Liu

**Affiliations:** 1 Department of Cardiovascular Surgery, the First Affiliated Hospital of Harbin Medical University, 23 Youzheng Road, Harbin, Heilongjiang 150001 China; 2 Department of Magnetic Resonance Imaging (MRI), the First Affiliated Hospital of Harbin Medical University, Harbin, China

## Abstract

**Introduction:**

To determine whether keeping the heart empty and beating is an effective technique to preserve hypertrophied pig hearts, and to investigate the underlying mechanism.

**Methods:**

Ten Bama Miniature pigs with hypertrophied hearts were divided into 2 groups (n = 5 in each group). One group underwent normothermic normokalemic simultaneous perfusion (NNSP). The other group was subjected to normothermic hypermokalemic simultaneous perfusion (NHSP) and used as controls. Cardiac contractive function, myocardial energy metabolism and myocardial perfusion were assessed using magnetic resonance imaging. Western blot analysis was carried out to determine the expression of Troponin I (cTnI), Troponin T (cTnT), SM-MHC, Casapase-3 and PARP4. TUNEL assay was used to detect apoptotic cardiomyocytes.

**Results:**

Keeping the heart empty and beating with NNSP improved the preservation of contractile function in comparison with cardioplegic arrest using NHSP. No significant differences existed in the effects of NNSP and NHSP in maintaining myocardial energy metabolism. 13 % perfusion defects areas were found in one heart in the NHSP group, whereas none was found in all other hearts in both groups. The expressions of cTnI, cTnT, Casapase-3 and PARP4 in NHSP group were abundantly increased compared to NNSP group as measured by Western blotting. Conversely, the expression of SM-MHC in NHSP group was reduced compared with NNSP group. The number of TUNEL positive nuclei per mm^2^ area was significantly increased in NHSP group compared with NNSP group.

**Conclusions:**

Keeping the heart beating with NNSP is an alternative technique to preserve hypertrophied hearts during valvular surgery.

## Background

It is well acknowledged that to date cardiac surgery is the most effective therapeutic treatment for valve diseases [1]. For safe and effective valve surgery, adequate myocardial protection is considered to be an important aspect. Hypothermic cardioplegia techniques were applied and well accepted to induce myocardial protective effects by reducing oxygen consumption in the initial stage [2]. However, it was observed later that cardioplegia techniques could cause subsequent cardiac impairment, especially reperfusion injury [3], especially in patients with compromised cardiac function, such as severe myocardial hypertrophy, low ejection fraction, previous coronary revascularization and multiple valve pathologies [4–8].

Myocardial hypertrophy, accompanied with the development of perivascular and interstitial accumulation of connective tissue, directly deteriorates contractility and relaxation of the heart [9–11]. It is also known to be associated with a significant enlargement of the myocardial interstitial compartment and a decrease in the density of the capillaries and mitochondria [12–14]. These pathologic changes have established the histologic foundation for severe and compromised myocardial perfusion and energy metabolism. When using cardioplegia techniques as myocardial protective methods, cardioplegia could abolish the squeezing effect of myocardial contraction on the coronary system, which is essential for maintenance of normal myocardial fluid homeostasis and blood perfusion [15, 16]. Besides, cardioplegia could lead to an increase in potassium and chloride, which may cause water accumulation in both the extracellular and intracellular compartments [17, 18]. Therefore, considering both pathologic changes in myocardial hypertrophy and the side effects of cardioplegia, cardioplegic techniques may not offer optimal protection for hypertrophied hearts in high-risk patients.

Since the development of beating coronary artery bypass grafting (CABG), cardioplegia is no longer considered to be an indispensable component for cardiac surgery [19]. Whether to keep the heart beating or not does not directly affect surgical precision. By extension, it is believed that valve surgery can also be performed under beating conditions. In the early days prior to the development of cardioplegia, beating heart valve surgery was the only available method for myocardial protection [20]. The published preliminary clinical experience suggested that keeping the heart empty and beating during valve surgery is conducive to high-risk patients [21, 22]. It is hypothesized that the potential beneficial effects of keeping the heart empty and beating are closely related to relieving the apoptosis of myocardial cell. In addition, It is also speculated that keeping the heart empty and beating could improve hypertrophy myocardial blood perfusion, contractile function and energy metabolism. This experiment is designed to verify whether the speculation can be met or not.

## Methods

All procedures involving animals were approved by Harbin Medical University Ethics Committee for Animal Experiments and performed in accordance with the Guide for the Care and Use of Laboratory Animals published by the US National Institutes of Health.

### Pig model of pressure-overloaded left ventricular hypertrophy

Ten 8- to 10-week-old Bama Miniature Pigs weighing 10 to 15 kg were sedated with an intramuscular injection of diazepam (0.4 mg/kg body weight) and ketamine (20 mg/kg body weight). Once anesthetized, piglets were intubated and mechanically ventilated. Intravenous injection of 3 % pentobarbital sodium was given to maintain anesthesia throughout the operation. To start, a left lateral thoracotomy was performed in the third intercostal space. The pericardium was incised, and care was taken to avoid damage to the phrenic nerve. A piece of suture inside a silicone tube was then placed to circle the ascending aorta. Followed by that the ends of the suture were tied to allow for overlap of the tube ends to create a peak systolic pressure gradient of 10 to 20 mm Hg between the left ventricle (LV) and aorta distal to the stenosis. The operation finished by closing the chests and the animals were allowed to recover for 12 weeks for the development of LV hypertrophy.

### The protection model of hypertrophy myocardium

Twelve weeks after aortic banding, the chests were reopened under a general anesthesia. Firstly the pericardium was opened longitudinally along the midline. After animal heparinization (3,000 IU heparin into a peripheral vein), the aorta, pulmonary artery, and inferior and superior vena cava were dissected and clamped. Conventional cardiopulmonary bypass was established with ascending aortic and single right atrial cannulation with systemic normothermia (36.5 °C-37 °C), perfusion flow rate 250 ml/min and retrograde perfusion pressure at 80-120 mmHg. An aspirator was placed near the coronary sinus through an incision under the right atrium. The effluent was collected continuously by the aspirator.

Pig blood was collected from the animal chest and mixed with Krebs-Henseleit solution in a 1:1 ratio for perfusion. Krebs-Henseleit solution is widely accepted as a physiologic perfusion medium and has been used for many years for heart perfusion. The Krebs-Henseleit solution contained 118 mmol/L NaCl, 1.2 mmol/L MgSO_4_, 0.5 mmol/L ethylenediaminetetraacetic acid, 11 mmol/L glucose, 25 mmol/L NaHCO_3_, 1.75 mmol/L CaCl_2_ and 0.625 % bovine serum albumin. The concentration of potassium was 4.0 and 16 mmol/L for normothermic normokalemic simultaneous perfusion (NNSP) and normothermic hyperkalemic simultaneous perfusion (NHSP), respectively. The temperature of the heart was maintained at 36.5 °C to 37 °C throughout the operation.

### Experimental protocols

Ten hypertrophied hearts were divided into two groups with five hearts in each group. Group 1 (n = 5) were used to evaluate the effect of empty beating (NNSP). Group 2 (n = 5) were used to evaluate the effect of cardioplegic arrest (NHSP). Both groups underwent a protocol consisting of 80 min of either NNSP (group 1) or NHSP (group 2). The 80-min preservation period was chosen because it was sufficient for most valve surgeries. Intermittent delivery was used for normothermic cardioplegic arrest in this study. After the 80-min preservation period, the aortic cross clamp was removed in both groups. Normal perfusion of was given and patients core temperature stayed 35–37 °C. The sternum was subsequently closed.

Cardiac contractive function and LV wall thickness were assessed using cine MRI immediately after the surgery. The total time interval was less than 20 min. Myocardial energy metabolism was monitored by using phosphorus 31 magnetic resonance spectroscopy. MR perfusion imaging was used to evaluate myocardial perfusion. All of the MRI images were collected by Philips Achieva 3.0 T superconducting MRI scanner.

After imaging examination, the animals were killed, autopsied and the myocardial specimens were harvested. Both the heart and LV weight (LV free wall and septum) were measured. Corresponding data of normal pig hearts, used as control for this study, was referred to another study of us which had not been published yet (data unpublished). Tissues of LV (with coronary artery) were taken for lab experiment. Western blot analysis was carried out to determine the expression of Troponin I (cTnI), Troponin T (cTnT), smooth muscle myosin heavy chain (SM-MHC), PARP4 and Casapase-3. TUNEL assay was used to detect apoptotic cardiomyocytes.

### Assessment of contractile function

Left ventricular end-systolic volume and left ventricular end-diastolic volume were measured by the cine MRI. Contractile ability of the hypertrophied hearts was also assessed by left ventricular ejection fraction [(left ventricular end-diastolic volume and left ventricular end systolic volume) / left ventricular end-diastolic volume].

### Phosphorus 31 MR spectroscopy

Phosphorus 31 (^31^P) MR spectroscopy was performed on a 3-T magnet equipped. The ^31^P MR spectra were acquired using a MR surface coil. Monitoring ^31^P MR signal intensity was performed using a point resolved spectral selection (PRESS) sequence during the test. Phosphorus compounds included phosphocreatine (PCr), inorganic phosphate (Pi) and 3 peaks (α, β and γ) of adenosine triphosphate (ATP) were observed and noted. The β peak was used for quantifying ATP.

### MR perfusion imaging

The blood perfusion of hypertrophy myocardial tissue was evaluated by MR perfusion imaging. Monitoring MR perfusion imaging signal intensity was performed using a echo-planer imaging (EPI) sequence. The pig hearts in groups 1 (*n* = 5) and 2 (*n* = 5) were subjected to the injection of Gd-DTPA (0.05 mmol/kg body weight; Magnevist, Berlex, Canada). 10 min after contrast agent was injected, late enhancement was performed using a phase sensitive inversion recovery (PSIR) sequence.

### Western blot

Freshly frozen myocardial tissue samples were homogenized in RIPA buffer. Protein from myocardium tissues was extracted and separated by sodium dodecyl sulfate-polyacrylamide gel electrophoresis (SDS-PAGE). Proteins was then transferred to nitrocellulose membranes, blocked and probed sequentially with primary antibodies against cTnI, cTnT, SM-MHC, PARP4 (Bioss Biotechnology, Inc) and Casapase-3 (Cell Signaling Technology, Inc). After incubation in the primary antibody, the membrane was incubated in an appropriate secondary antibody. After washing, the bound antibody complexes were detected using an electrochemiluminescence reagent.

### Apoptosis detection and quantification by TUNEL assay

Detection of apoptosis was carried out by a terminal deoxynucleotidyl transferase (TdT) dUTP nick-end labelling (TUNEL) assay (Roche Diagnostics GmbH). Approximately 20 randomly chosen, non-overlapping images covering the infarct and border regions were acquired using a × 200 objective. Blinded observers counted the number of TUNEL positive nuclei in each image. The result was then expressed as the number of TUNEL positive nuclei per mm^2^ area.

### Data analysis

All the data collected from MRI were analyzed by using the cardiac analysis package in the post-processing workstation. All numerical results were expressed as the mean ± S.D. of the mean. Student’s *t* test (unpaired) was used to analyze the differences between NHSP and NNSP. A value of *P* less than 0.05 indicates significant difference.

## Results

### Myocardial hypertrophy

The weight ratio of LV to entire heart was significantly (*P* < 0.05) higher in hypertrophied hearts (0.58 ± 0.04) than in normal hearts (0.48 ± 0.03). The LV wall of the hypertrophied hearts was significantly (*P* < 0.05) thicker (2.02 ± 0.17 cm) than that of the normal hearts (1.24 ± 0.05 cm). These data clearly indicates that 12-week aortic banding has resulted in significant LV hypertrophy.

### Effect of NNSP and NHSP on contractile function

**Fig. 1:**
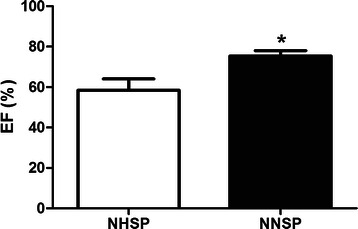
Comparison of EF between the NHSP hearts and NNSP hearts. Averages of EF measurements were lower during NHSP than during NNSP. Abbreviations: NHSP, normothermic hyperkalemic simultaneous blood perfusion; NNSP, normothermic normokalemic simultaneous blood perfusion; EF, ejection fraction

After undergoing NNSP and NHSP, Left ventricular end-systolic volume and left ventricular end-diastolic volume of both groups were measured by the cine MR imaging. Contractile ability of the hypertrophied hearts was assessed by left ventricular ejection fraction [(left ventricular end-diastolic volume and left ventricular end-systolic volume)/left ventricular end-diastolic volume]. As the result shown in Fig. 1, averages of left ventricular ejection fraction measured in NHSP (58.43 ± 5.615 %) were lower than in NNSP(75.33 ± 2.675 %).

### Efficacy of NNSP and NHSP for sustaining myocardial energy metabolism

**Fig. 2:**
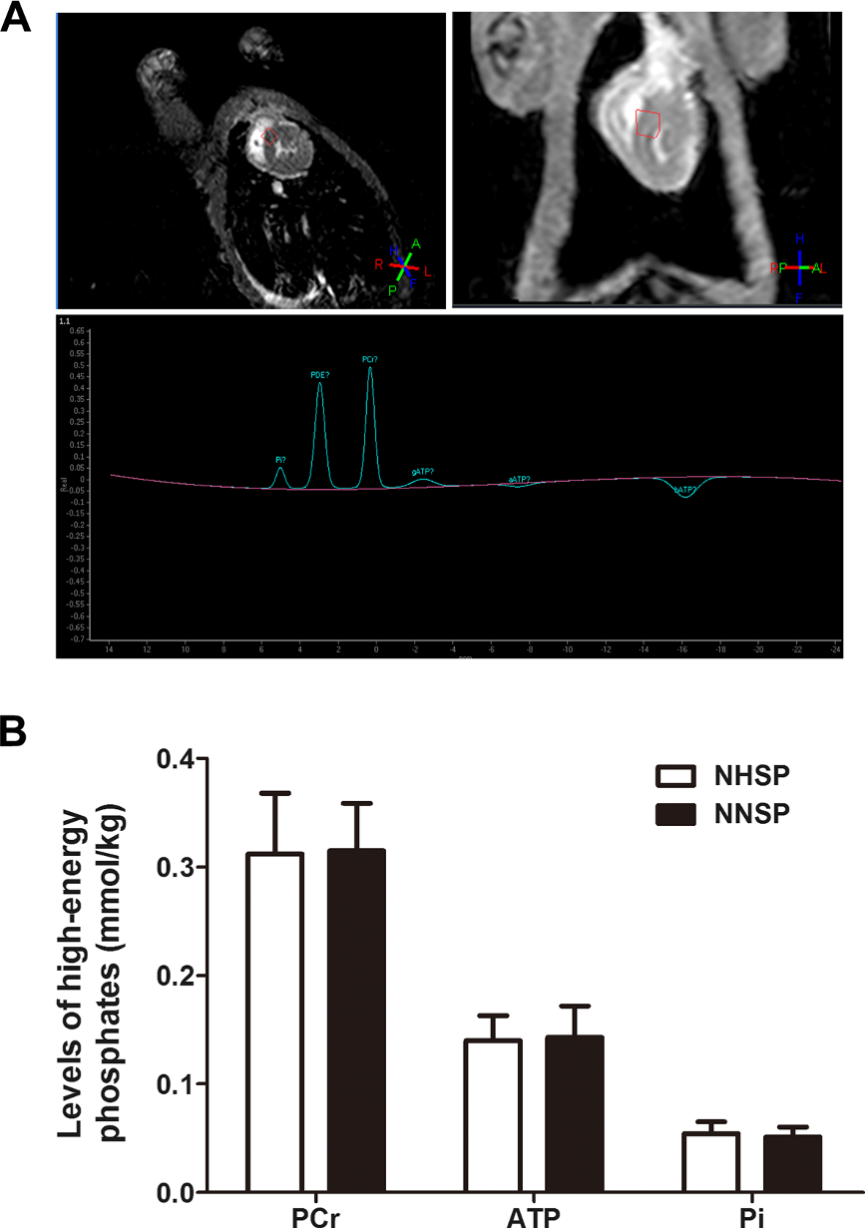
An example of obtaining ^31^P MR spectra (**A**). Averaged levels of the myocardial high-energy phosphates Pi, PCr and ATP measured during NHSP and NNSP (**B**). No significant changes in Pi, PCr, or ATP were observed in the both group. Abbreviations: Pi, inorganic phosphate; PCr, phosphocreatine; ATP, adenosine triphosphate; NHSP, normothermic hyperkalemic simultaneous blood perfusion; NNSP, normothermic normokalemic simultaneous blood perfusion.MR, magnetic resonance; ^31^P, phosphorus 31

Representative ^31^P MR spectra obtained from NNSP and NHSP hearts are shown in Fig. 2A. Average levels of myocardial PCr, Pi and ATP are shown in Fig. 2B. No significant changes were caused in the levels of myocardial ATP, PCr and Pi by either perfusion techniques.

### Effect of NNSP and NHSP on the blood perfusion of hypertrophy myocardial tissue

MR perfusion imaging was used to evaluate the effect of NNSP and NHSP on the blood perfusion of hypertrophy myocardial tissues. The aim of perfusion scanning is to identify ischemia areas, which are seen as perfusion defects areas by MR perfusion imaging. No ischemia areas were found in NNSP group. In the NHSP group one particular heart however was found to have 13 % perfusion defects areas, while all others in the same group appear to have none.

### Effect of NNSP and NHSP on the degree of myocardial fibers protein hydrolysis and blood vessel damage

**Fig. 3:**
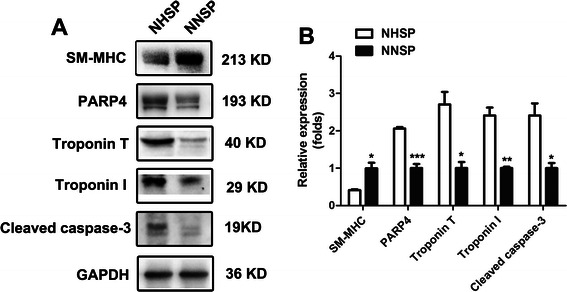
Representative Western blots (**A**) and the results of quantitative analysis (**B**) of LV myocardial tissue after operations. Western blot analysis was carried out to determine the expression of cTnI, cTnT, SM-MHC, PARP4 and Casapase-3. GAPDH was used as a control reference. Data are reported as mean ± S.D. for three independent experiments (*, *P* < 0.05, **, *P* < 0.01, ***, *P* < 0.001; unpaired Student’s *t* test). Abbreviations: cTnI, Troponin I; cTnT, Troponin T; SM-MHC, smooth muscle myosin heavy chain; NHSP, normothermic hyperkalemic simultaneous blood perfusion; NNSP, normothermic normokalemic simultaneous blood perfusion

In order to explore the effect of NNSP and NHSP have on the degree of myocardial fibers protein hydrolysis and blood vessel damage, western blot analysis was carried out to determine the expression of cTnI, cTnT and SM-MHC. As shown in Fig. 3, the expression level of cTnI and cTnT in NHSP group were significantly increased in the heart compared with NNSP group (*P* = 0.0027 and *P* = 0.0104, respectively). On the contrary, the expression of SM-MHC in NHSP group was significantly reduced compared with NNSP group (*P* = 0.0166).

### Effect of NNSP and NHSP on the protein expressions of Caspase-3 and PARP4

To investigate the cardiac damage following NNSP and NHSP, the levels of the apoptosis-regulatory proteins including PARP4 and Casapase-3 was evaluated by western blot analysis. As shown in Fig. 3, the protein expression of caspase-3 in NHSP group was abundantly increased in the heart compared with NNSP group (*P* = 0.0163). Additionally, PARP4 was also considered to play a pivotal role in the mediation of apoptotic cascades. Our results revealed a dramatically elevated expression level of PARP4 in the heart tissue of NHSP group in comparison to NNSP group (*P* = 0.0008) (Fig. 3).

### Effect of NNSP and NHSP on apoptosis

**Fig. 4:**
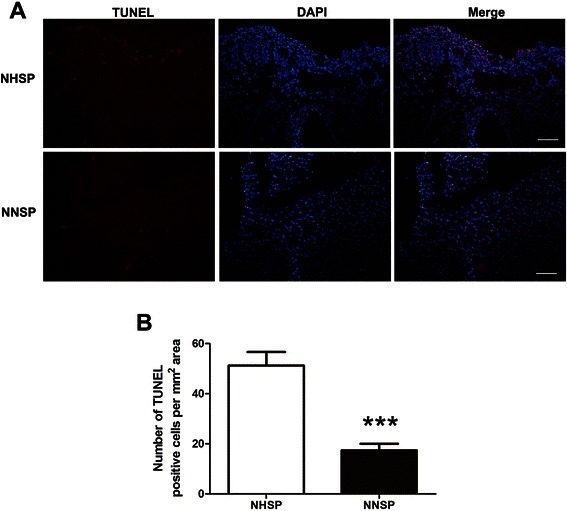
Myocardial apoptosis was detected by TUNEL staining. **A** The apoptotic cells were indicated by TUNEL-positive nuclei (red staining). **B** Quantification of the number of TUNEL positive nuclei per mm^2^ area. Data are reported as mean ± S.D. for three independent experiments (***, *P* < 0.001; unpaired Student’s *t* test). Abbreviations: NHSP, normothermic hyperkalemic simultaneous blood perfusion; NNSP, normothermic normokalemic simultaneous blood perfusion

Detection and quantification of apoptosis were carried out by TUNEL assay. As shown in Fig. 4, the number of TUNEL positive nuclei per mm^2^ area was significantly (*P* < 0.0001) increased in NHSP group (51.22 ± 5.389) as compared with NNSP group (17.40 ± 2.601).

## Discussion

Cardiac surgery has been considered to be a reliable treatment to correct valve abnormalities and to alleviate the life-threatening symptoms and complications of valve diseases [1]. Myocardial protection is an important aspect of safe and effective valve surgery. At present, cardioplegic techniques provide myocardial protection. However, even with continuous warm blood cardioplegia, which is considered to be the best form of myocardial protection [23, 24], some degree of postoperative myocardial dysfunction may still occur [25]. This suggests cardioplegic techniques may only be the second best choice for protection, especially for hypertrophied hearts [4–8]. The detrimental effects of cardioplegia on contractile function, energy metabolism, myocardial blood perfusion and the apoptosis of myocardial cell may directly lead to postoperative cardiac dysfunction. The published preliminary clinical experience suggests that keeping the heart empty and beating during valve surgery is conducive to high-risk patients [21, 22]. This study was performed to evaluate the effects of this technique on contractile function, energy metabolism, myocardial blood perfusion and the apoptosis of myocardial cell.

Research has shown that cardioplegia techniques cause cardiac impairment, especially ischemia-reperfusion injury [2]. Such injury leads to degradation of troponin I and reduction of isotonic contraction of muscle force [26]. These changes inhibit myocardial contractile function [27]. Therefore, the inhibition of myocardial contractile function was expected to be more severe in NHSP than in NNSP. In this study, it was found that the averages of left ventricular ejection fraction measured from the NHSP hearts were significantly lower than those obtained from the NNSP hearts. It is a fact that cardioplegia can pause the electromechanical activity of the heart and reduces the myocardial energy demand by a factor of approximately 100 [28]. In this particular study, it was observed that hearts subjected to NHSP did not show any significant change in the levels of ATP and PCr compared to those subjected to NNSP. Thus, it can be concluded that the effects of NNSP and NHSP in maintaining myocardial energy metabolism were not significantly different; this was anticipated.

The aim of perfusion scanning is to identify ischemia areas. In this study, ischemia areas were found in one heart in the NHSP group only. This observation however is not sufficient enough to conclude the effect of NNSP and NHSP on the blood perfusion of hypertrophy myocardial tissue. Therefore, further investigation is still required to determine the effect of keeping the heart empty and beating on myocardial perfusion.

McDonough and Gao WD found that ischemia reperfusion injury caused degradation of cTnI and reduction of isotonic contraction of muscle force [26, 29]. The degradation of cTnI inhibited myocardial contractile function [27]. Ischemia reperfusion injury was one of the cardiac impairment of cardioplegia techniques [2]. The expression of cTnI in NHSP group was abundantly increased in the heart compared with NNSP group. From the result, it seems that NNSP has the ability to lessen the degree of myocardial fibers protein hydrolysis and phosphorylation.

cTnT is a contractile protein that is rarely found but only present when myocardial necrosis occurs [30]. As an important parameter for assessing the cardiac injury in ischemic heart diseases, cTnT data was garthered through western blot analysis in this study. From the result, it is clearly illustrated that the activities of cTnT were evidently increased in the NHSP group than those in the NNSP group. This suggested that the cardiac injury caused by NHSP was more severe than by NNSP.

As one of the composition of contraction, the expression of SM-MHC decreases when the blood vessels were found to be damaged [31]. As revealed, reductions in the expression of SM-MHC in both groups can be seen. In comparison however, the NHSP group experienced a greater reduction. Thus, it can be speculated that the hearts in the NHSP group had more serious blood vessel damage than those in the NNSP group.

Cardioplegia techniques and ischemia reperfusion injury has led to an increased apoptosis-regulatory proteins (Caspase-3 and PARP 4) activity. The proteins activated the apoptosis signaling pathways. So, cardioplegia techniques and ischemia reperfusion injury were considered to be the main cause of myocardial cell apoptosis [32, 33]. The activity of Caspase-3 and PARP 4 were increased in the NHSP group compared to those in the NNSP group. Therefore, NHSP caused more serious of myocardial cell apoptosis. In addition, the result of TUNEL assay also proved this.

In this study, since histologic assessment of the hypertrophied heart was not performed. No histologic evidence of myocardial hypertrophy was provided. Its pathologic changes might be somewhat different from those of a human’s hearts. However, LV wall thickness and heart weight data indicated significant myocardial hypertrophy. Results from this study have demonstrated that keeping the heart empty and beating with normothermic normokalemic simultaneous blood perfusion improved the preservation of contractile function in comparison with cardioplegic arrest using normothermic hyperkalemic blood perfusion. The basic study was proved in protecting myocardial injury, blood vessel damage and myocardial apoptosis, keeping the heart empty and beating with normothermic normokalemic simultaneous blood perfusion has obvious advantages.

Although our results suggest that keeping heart empty and beating provides better mycardial protection than traditional cardioplegia techniques, our study still suffers from some limitations. Firstly, we only used one kind of infusion solution (Krebs-Henseleit solution). With the development of cardiac surgery, various infusion solutions have been developed. Further research should be carried out to compare the effects of different infusion solutions and select an optimal one. Secondly, we only detected the level of biomarkers for myocardial injury (cTnI and cTnT) in myocardial specimens harvested after the surgery. The measurement of released biomarkers (e.g. Troponins or CKMB) from the heart during reperfusion is also an important indicator for myocardial injury. Further study should also measure the released biomarkers for cardiac injury in circulation.

## Conclusions

In summary, compared to cardioplegic arrest using NHSP, keeping the heart beating with NNSP has improved preservation for hypertrophied hearts. It is also noted that further investigation is required to determine the effects of keeping the heart empty and beating on myocardial perfusion. Keeping the heart beating with NNSP is an alternative technique to preserve hypertrophied hearts during valvular surgery.

## Abbreviations

NNSP: Normothermic normokalemic simultaneous perfusion
NHSP: Normothermic hypermokalemic simultaneous perfusion
CPB: Cardiopulmonary bypass
CABG: Coronary artery bypass grafting
LV: Left ventricle
K-H: Krebs-Henseleit
EPI: Echo-planer imaging
PSIR: Phase sensitive inversion recovery
